# Machine learning-based analysis of factors influencing surgical duration in type A aortic dissection

**DOI:** 10.3389/fpubh.2025.1682339

**Published:** 2025-10-31

**Authors:** Dechao Deng, Xiaoming Zhang, Xiangzhen Feng, Gaoli Liu, Pingping Wang, Jinyu Cong, Xiang Li, Kunmeng Liu, Benzheng Wei

**Affiliations:** ^1^Center for Medical Artificial Intelligence, Shandong University of Traditional Chinese Medicine, Qingdao, China; ^2^Qingdao Academy of Chinese Medical Sciences, Shandong University of Traditional Chinese Medicine, Qingdao, China; ^3^Qingdao Key Laboratory of Artificial Intelligence Technology for Chinese Medicine, Qingdao, China; ^4^Department of Cardiovascular Surgery, The Affiliated Hospital of Qingdao University, Qingdao, China

**Keywords:** Stanford Type A aortic dissection, machine learning, prediction models, surgical duration, SHapley Additive exPlanations

## Abstract

**Background:**

Stanford Type A aortic dissection (TAAD) is a life-threatening condition involving the ascending aorta and requires urgent surgery. This study developed 11 machine learning regression models to predict operative duration and identify key clinical factors influencing surgical time in TAAD.

**Materials and methods:**

In this single-center retrospective cohort study of 505 patients who underwent surgery from December 2017 to March 2023. Specifically, 11 machine learning models were construct using 47 preoperative and intraoperative features to predict operative duration. Model performance was assessed by R^2^, RMSE, and MAE, and SHAP analysis enhanced interpretability.

**Results:**

The study primarily consisted of middle-aged patients, comprising 73.4% males and 26.6% females. Furthermore, most patients underwent complex aortic procedures under time-constrained preoperative conditions. Procedures involving root replacement and total arch replacement were associated with longer surgical durations. The ExtraTrees Regressor had the highest predictive accuracy. SHAP analysis revealed five key features: Duration of extracorporeal circulation, Duration of aortic occlusion, Intraoperative blood transfusion, Treatment method for the aortic arch, and Treatment method for the aortic root.

**Conclusion:**

This study developed high-performance predictive models to identify key features affecting operative duration in TAAD surgery. Complex reconstructions prolong procedures, and longer aortic occlusion further contributes to this effect. The findings highlight the major influence of surgical strategies and intraoperative management on surgical duration. Special consideration remains warranted for specific patient subgroups.

## 1 Introduction

Aortic dissection (AD) is a catastrophic vascular emergency initiated by a tear in the aortic intima, through which blood enters the medial layer, creating a false lumen and leading to separation of the aortic wall layers (1). This condition necessitates prompt medical and often surgical intervention (2). A previous study showed that the weighted annual incidence of aortic dissection at approximately 2.79 cases per 100,000 people (3). Among patients with untreated acute aortic dissection, the mortality rate increases by approximately 1–2% per hour after symptom onset (4), with in-hospital mortality reaching up to 52% in those who do not undergo surgical intervention (5). Even with treatment, the 5-year survival rate for patients with acute aortic dissection ranging from 45 to 88% (6). AD poses a significant clinical challenge in critical care medicine, requiring a multidisciplinary response and intervention. The Stanford classification system categorizes AD into Type A (involving the ascending aorta) and Type B (not involving the ascending aorta) (7). Stanford Type A aortic dissection (TAAD) is more severe and typically necessitates urgent open-chest surgery for aortic replacement and reconstruction (8). Due to the extensive nature of the procedure, which involves cardiac and cerebral perfusion management, TAAD is associated with a substantially higher frequency of sudden events during surgery and elevated mortality compared to Type B (9, 10).

In TAAD surgery, intraoperative parameters such as surgical duration and the duration of extracorporeal circulation are closely associated with patient outcomes. Studies have identified surgical duration, duration of extracorporeal circulation, and aortic cross-clamp time as independent risk factors for postoperative mortality (11). Surgical duration is also directly linked to resource utilization. Longer anesthesia and extended cardiopulmonary bypass time often lead to extended surgical durations. This may require prolonged postoperative ICU monitoring, which increases the consumption of medical resources and overall healthcare costs (12, 13). These factors underscore the importance of tightly controlling surgical duration to improve patient outcomes and optimize hospital resource allocation. Therefore, the critical importance of accurately predicting surgical duration for clinical management, reliable prediction remains challenging in current clinical practice (14). Surgical duration is influenced by numerous factors, including patient-specific characteristics, procedural complexity, and intraoperative events (15). The previous studies have demonstrated that adverse environmental conditions are significant triggers for acute cardiovascular events, including aortic dissection (16, 17). From this broader pathophysiological perspective, patients presenting under such conditions may arrive in a more severe or unstable baseline state, which can indirectly increase procedural complexity and operative duration. Moreover, traditional estimation methods based on surgeon experience or historical averages often lack precision and are subject to considerable variability (18).

In recent years, increasing attention has been given to the integration of clinical research and machine learning, reflecting a broader trend toward combining data-driven algorithms with medical practice to improve prediction, evaluation, and decision-making in healthcare (19–21). Recent work in machine learning-based operative prediction has shown promising results in forecasting postoperative outcomes in cardiac surgery, including mortality, complications, and ICU stays (22–24). Furthermore, cutting-edge ML approaches have been increasingly applied to optimize cardiovascular surgical strategies and perioperative decision-making using multimodal clinical data, highlighting their potential to refine risk stratification and improve surgical outcomes (25–27). Clinical studies have highlighted that operative duration in aortic surgery is influenced by multiple perioperative factors, including aneurysm diameter, extent of reconstruction, and perfusion strategy, which are consistently linked with prolonged cardiopulmonary bypass and ischemic times (22, 28, 29). Furthermore, recent work has developed prognostic tools and risk models that incorporate intraoperative variables to better capture predictors of prolonged surgery and adverse outcomes in aortic and major cardiovascular operations (26, 30).

As a highly complex cardiovascular procedure, TAAD surgery is both time-consuming and technically demanding. However, current clinical research lacks an in-depth analysis of the specific factors influencing its operative duration. The purpose of this study is to comprehensively analyze preoperative and intraoperative factors associated with surgical duration using machine learning. Both preoperative and intraoperative variables were included to reflect the overall influencing factors of operation duration. Accurate prediction of surgical duration can facilitate efficient operating room scheduling, optimize anesthesia and cardiopulmonary bypass management, enhance surgical safety, and reduce healthcare costs.

## 2 Materials and methods

### 2.1 Informed consent

This study was approved by the Ethics Committee of the Affiliated Hospital of Qingdao University (No. QDFY WZLL 29835). Data were collected between December 2017 and March 2023, and written informed consent was obtained from all participants.

### 2.2 Data collection

This single-center retrospective cohort included 675 consecutive patients who underwent surgery for acute type A aortic dissection at the Affiliated Hospital of Qingdao University between December 2017 and March 2023. Two complementary approaches were employed to justify the sample size. Firstly, based on a precision-based sample size calculation derived from our dataset with a desired 95% confidence interval half-width of 15 min, the minimum required sample size was estimated at 311 patients. The detailed formula used for this calculation is provided in the Supplementary materials. As our study included 675 patients, the available sample size substantially exceeded this requirement, thereby ensuring adequate statistical precision (31). Secondly, given the aim of analyzing factors associated with operative duration, this study included 47 features and justified the sample size by considering expected effect size (32). Following Green’s rules of thumb, at least 50 + 8 m observations are recommended for testing the overall model and at least 104 + m for testing an individual regression coefficient, where m denotes the number of features (33). In this study (m = 47), the sample size of 675 patients substantially exceeded the corresponding thresholds (426 and 151). Furthermore, compared with other similar studies, the enrolled sample size in this study can be considered appropriate (34, 35).

This study identified candidate predictor variables for potential inclusion in our models from a review of the literature (16, 17, 36–38). In addition to clinical features, climate features were included in this study to explore the potential influence of environmental factors on operative duration. All 47 features were shown as follows: (a) preoperative features, Surgery Preparation Time, ICU stay days before surgery, Gender, Age, Height, Weight, Heart rate, Body temperature, Systolic blood pressure, Diastolic blood pressure, Hypertension, Diabetes, Heart disease, Lung disease, Marfan syndrome, Smoking quantity, Alcohol consumption, Loss of consciousness, White blood cells, Red blood cell count, Hemoglobin, Platelets, C-reactive protein, Albumin, Alanine transaminase, Aspartate transaminase, Creatinine, Troponin I or T, D-dimer, Minimum temperature, Maximum temperature, Relative humidity, Air Quality Index (AQI), Fine particulate matter (PM2.5), Coarse particulate matter (PM10), Carbon Monoxide (CO), Nitrogen dioxide (NO₂), Sulfur dioxide (SO₂), 8-h average Ozone concentration (O₃₋₈h). (b) intraoperative features, Treatment method for the aortic root, Treatment method for the aortic arch, Intraoperative blood transfusion, Duration of extracorporeal circulation, Duration of aortic occlusion, Duration of deep low-temperature shutdown cycle, Duration of ventilator use, Aortic valve insufficiency. To ensure clarity and reproducibility, all variables used in the analysis and their corresponding units are summarized in Supplementary Table S1.

### 2.3 Data cleaning

**Figure 1:**
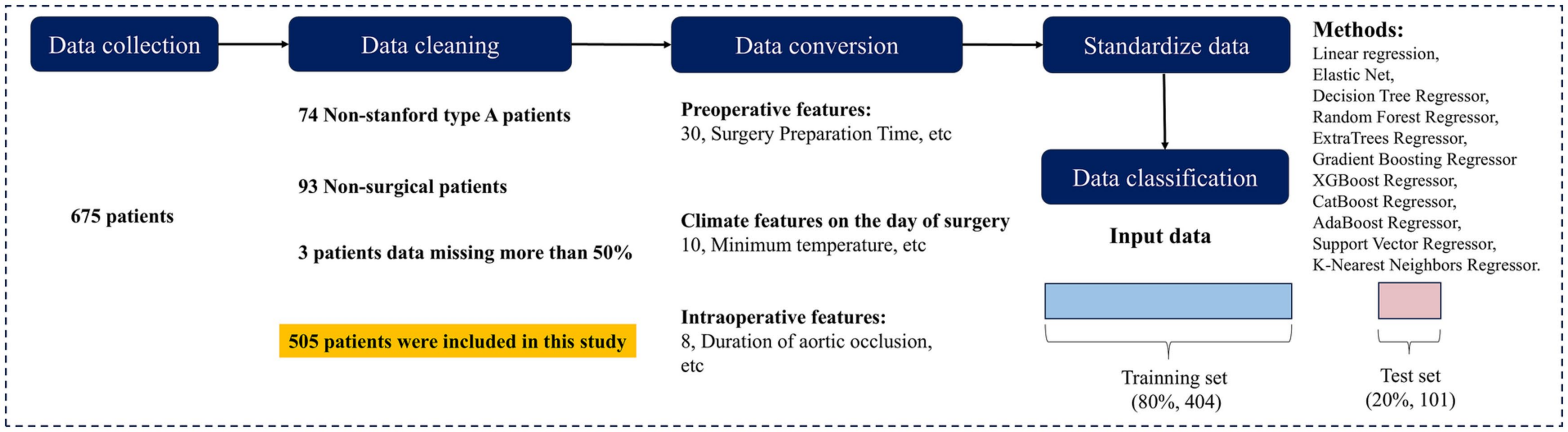
Data collection and preprocessing.

To ensure data accuracy and completeness, data cleaning was conducted in accordance with the study objectives. A total of 675 patients diagnosed with AD were initially screened. After excluding 74 patients with non-TAAD, 93 patients who did not undergo surgery, and 3 patients with more than 50% missing data, 505 cases were included in the final analysis. Baseline information of excluded patients is provided in Supplementary Table S2. The data selection process is illustrated in Figure 1.

### 2.4 Data processing

**Figure 2:**
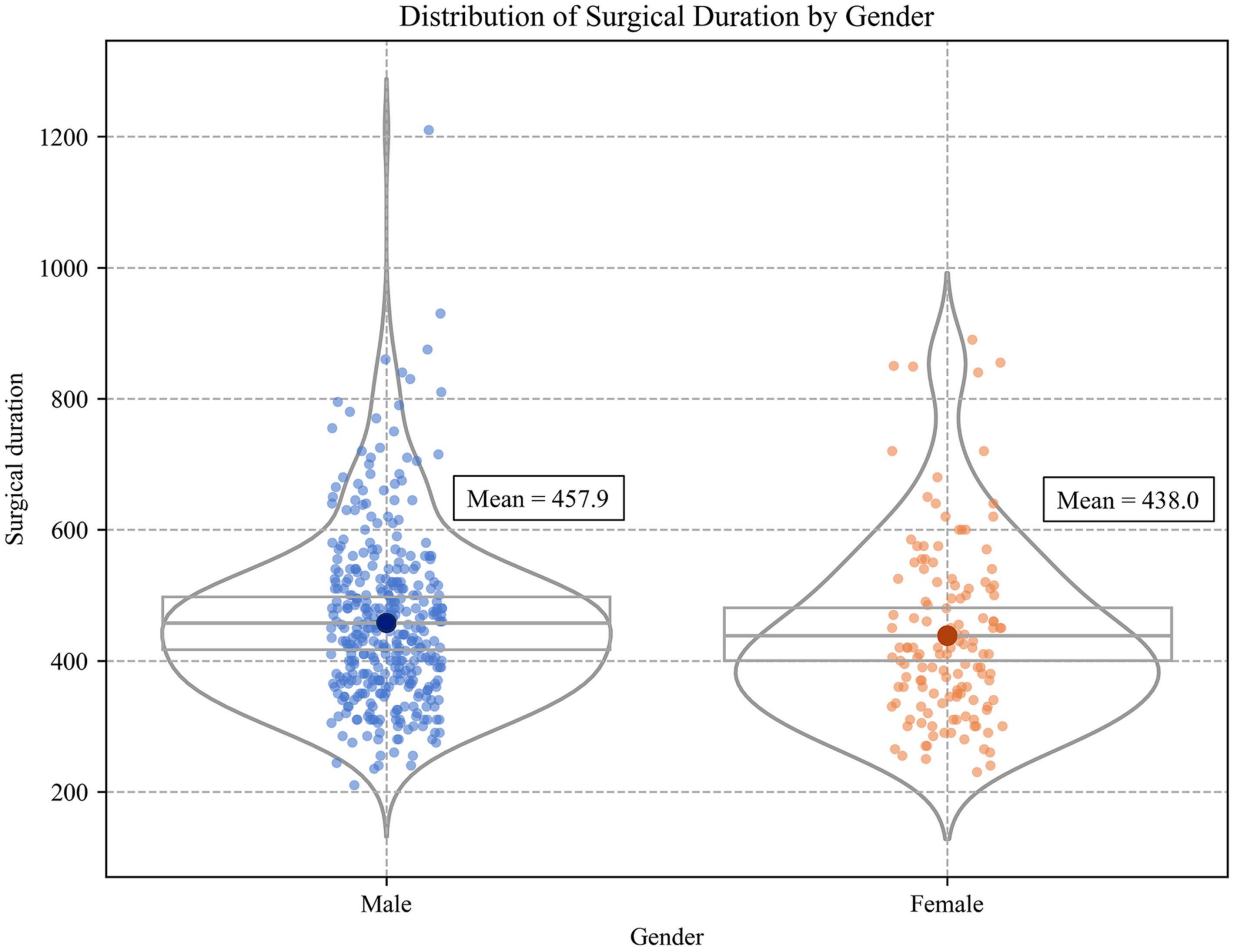
Distribution of surgical duration by gender.

Continuous variables were standardized, and missing values were imputed using Multiple Imputation by Chained Equations. The distribution of the outcome variable was examined and found to be well-balanced. Therefore, no additional resampling or correction for class imbalance was required. This method constructs a regression model for each missing variable, uses other variables as predictive factors, and iteratively fills in missing values multiple times to obtain relatively stable and reasonable interpolation results (39) (Figure 2).

The dataset was randomly split into training (70%), testing (20%), and validation (10%) subsets using a fixed random seed (42).

### 2.5 Statistical methods

For all clinical features, statistical comparisons were conducted according to variable type. Specifically, categorical variables with two levels were analyzed using the independent samples *t*-test, continuous variables were evaluated for linear correlation with operative duration using Pearson’s correlation analysis, and categorical variables with more than two levels were assessed by one-way analysis of variance (ANOVA). A two-tailed *p* < 0.05 was considered statistically significant.

### 2.6 Regression models

To accurately predict surgical duration in TAAD surgery, this study developed and compared 11 representative machine learning regression models, encompassing a range of approaches including linear regression, regularized regression, tree-based models, ensemble learning methods, and kernel-based techniques. All analyses were performed in Python (version 3.8) using scikit-learn (version 1.4.1 post1).

*Linear regression:* linear regression fits a linear relationship between input features and the target variable. It is easy to interpret but sensitive to outliers and assumes linearity among variables.

*Elastic net:* elastic net combines both L1 and L2 regularization to perform variable selection while maintaining model stability. It performs well when dealing with correlated features.

*Decision tree regressor:* the decision tree regressor splits the feature space into regions based on feature thresholds to predict continuous outcomes. It captures nonlinear relationships but is prone to overfitting.

*Random forest regressor:* random forest builds an ensemble of decision trees and averages their outputs to improve prediction accuracy. It is robust against overfitting and effectively handles high-dimensional data.

*ExtraTrees regressor:* ExtraTrees is similar to random forest but introduces more randomness in the splitting process by selecting thresholds at random. This increases training speed and reduces variance.

*Gradient boosting regressor:* the gradient boosting algorithm sequentially trains weak learners to correct residual errors of previous learners. It offers strong predictive power but is sensitive to parameter tuning and noise.

*XGBoost Regressor:* XGBoost is an optimized gradient boosting algorithm that incorporates regularization and efficiently handles missing data. It is widely adopted due to its high speed and accuracy.

*CatBoost regressor:* CatBoost is designed to handle categorical variables efficiently and mitigate prediction shift during training. It offers enhanced stability and performance over traditional boosting methods.

*AdaBoost regressor:* AdaBoost combines multiple weak learners in a sequential manner, assigning higher weights to previously mispredicted samples. It performs well on clean datasets with low noise.

*Support vector regressor:* SVR constructs a hyperplane that fits the data within a defined margin of tolerance, based on support vector machine principles. It is suitable for small-to-medium-sized datasets and models nonlinear relationships.

*K-Nearest neighbors regressor:* KNN regressor predicts target values by averaging the outputs of the K most similar training samples. It is simple to implement but sensitive to feature scaling and less effective in high-dimensional spaces.

Hyperparameters for all models were optimized using a grid search. Linear Regression was fitted without regularization. For tree-based models (Decision Tree, Random Forest, ExtraTrees), parameters such as max_depth, min_samples_leaf, min_samples_split, and n_estimators were tuned. For boosting models (Gradient Boosting, XGBoost, CatBoost, AdaBoost), tuning focused on learning_rate, n_estimators, and max_depth, with additional regularization parameters included. For Elastic Net, the mixing parameter and regularization strength were optimized. For SVR, the penalty parameter and kernel parameters were tuned. For KNN, the number of neighbors and distance metric were considered. Model performance was evaluated using the coefficient of determination (R^2^), mean absolute error (MAE), and root mean squared error (RMSE), consistent with established practice in regression-based prediction.

Model performance was evaluated using a 5-fold cross-validation procedure. The dataset was randomly divided into five approximately equal subsets. In each iteration, four folds were used for training and the remaining fold was used for testing, such that every sample was evaluated exactly once in an independent test set. Performance metrics (R^2^, MAE, and RMSE) were recorded for each fold to provide an unbiased estimate of the model’s generalization ability on unseen data. This approach was selected as a balance between computational efficiency and robustness of performance estimation for a cohort of this size (*n =* 505).

*Coefficient of determination (R^2^):* R^2^ measures the proportion of the variance in the dependent variable that is predictable from the independent variables. Its value ranges from −∞ to 1, where a value closer to 1 indicates better model performance.


$$R^{2}=1-\frac{\sum_{i=1}^{n}{\left(y_{i}-{\hat{y}}_{i}\right)}^{2}}{\sum_{i=1}^{n}{\left(y_{i}-\bar{y}\right)}^{2}}$$

where $y_{i}\;$is the observed value, ${\hat{y}}_{i}$​ is the predicted value, and $\bar{y}\;$is the mean of the observed values.

*Root mean squared error (RMSE):* RMSE reflects the standard deviation of prediction errors and penalizes larger errors more severely. A lower RMSE indicates a better fit.


$$\text{RMSE}=\sqrt{\frac{1}{n}\sum_{i=1}^{n}{\left(y_{i}-{\hat{y}}_{i}\right)}^{2}}$$

*Mean absolute error (MAE):* MAE quantifies the average magnitude of errors between predicted and actual values, without considering their direction.


$$\mathrm{MAE}=\frac{1}{n}\sum_{i=1}^{n}|y_{i}-{\hat{y}}_{i}|$$

### 2.7 Code and data availability

The Python programming codes used for data preprocessing, model development, and generation of study outputs are publicly accessible through an open-access repository at https://github.com/ddc1103274511/TAAD.git. The dataset used in this study is available from the corresponding author upon reasonable request and with approval from the Ethics Committee of Qingdao University.

## 3 Results

### 3.1 Demographic characteristics

A total of 505 patients with TAAD were included in this study, as summarized in Table 1. The majority of patients were male (73.4%), with a mean age of 54.0 years (SD = 12.6). The average height and weight were 170.1 cm (SD = 10.9) and 77.4 kg (SD = 15.3). Preoperative vital signs showed a mean heart rate of 80.9 beats per minute (SD = 18.1), a mean systolic blood pressure of 134.4 mmHg (SD = 68.8), a mean diastolic blood pressure of 69.0 mmHg (SD = 17.1), and a mean body temperature of 36.5 °C (SD = 0.4). In terms of medical history, the prevalence of hypertension was 61.1%, cardiovascular disease 93.4%, and diabetes mellitus 98.0%. Laboratory findings indicated a generally elevated inflammatory response. The mean white blood cell count was 12.0 × 10^9^/L (SD = 4.2), the mean C-reactive protein level was 38.6 mg/L (SD = 41.1), and the mean D-dimer concentration was 6752.0 μg/L FEU (SD = 8437.4), suggesting that most patients exhibited a pronounced systemic inflammatory response and hypercoagulable state before surgery. In terms of surgical preparation and environmental factors, patients had a short preoperative preparation period, with a mean time from admission to surgery of 0.8 days (SD = 2.4), and a mean preoperative ICU stay of 0.6 days (SD = 1.6), reflecting that most procedures were performed as emergencies. To assess the potential impact of environmental stressors on surgery, preoperative external climate variables including ambient temperature, humidity, PM2.5, and AQI were also collected. In terms of surgical approach, 57.2% of patients underwent root replacement combined with ascending aorta procedures (‘root upgrade + replacement’), and 90.9% underwent total arch replacement, indicating the complexity of TAAD surgical procedures. The overall mean operative time for TAAD surgery was 452.7 min with a standard deviation (SD) of 134.9 min.

**Table 1:** Patient data involved in the study (including demographic, clinical, and climatic characteristics).

Characteristics	Mean/count (±SD/%)	*P*-value
Demographics		
Gender		0.13
Male	371 (73.4%)	
Female	134 (26.5%)	
Age	54.0 (±12.6)	0.0096
Height (cm)	170.1 (±10.9)	0.054
Weight (kg)	77.4 (±15.3)	0.023
Heart rate (bpm)	80.9 (±18.1)	0.534
Body temperature (°C)	36.5 (±0.4)	0.299
Systolic blood pressure (mmHg)	134.4 (±68.8)	0.504
Diastolic blood pressure (mmHg)	69.0 (±17.1)	0.507
Comorbidities		
Hypertension	309 (61.1%)	0.865
Diabetes	495 (98.0%)	0.396
Heart disease	472 (93.4%)	0.905
Lung disease	141 (27.9%)	0.0129
Marfan syndrome	5 (0.9%)	0.396
Loss of consciousness	37 (7.3%)	0.00038
Aortic valve insufficiency		<0.001
Mild	302 (59.8%)	
Mild to Moderate	7 (1.4%)	
Moderate	10 (2.0%)	
Moderate to Severe	4 (0.8%)	
Severe	182 (59.8%)	
Laboratory		
White blood cells (×10^9^/L)	12.0 (±4.2)	0.0048
Red blood cells (×10^9^/L)	4.2 (±1.5)	0.850
Hemoglobin (g/L)	125.5 (±20.2)	0.0080
Platelets (×10^9^/L)	164.4 (±59.1)	0.275
C-reactive protein (mg/L)	38.6 (±41.1)	0.030
Albumin (g/L)	37.2 (±4.7)	0.0126
Alanine transaminase (U/L)	85.6 (±545.1)	0.410
Aspartate transaminase (U/L)	115.7 (±600.1)	0.567
Creatinine (umol/L)	110.2 (±97.3)	0.291
Troponin I or T (ng/mL)	0.52 (±2.2)	0.044
D-dimer (μg/L)	6752.0 (±8437.4)	0.00022
Lifestyle Factors		
Smoking history	162 (32%)	0.422
Drinking history	88 (17.4%)	0.411
Surgical related data		
Surgery Preparation Time (day)	0.8 (±2.4)	0.016
ICU stay days before surgery (day)	0.6 (±1.6)	0.168
Minimum temperature (°C)	9.7 (±8.8)	0.191
Maximum temperature (°C)	15.3 (±8.9)	0.131
Relative humidity	0.7 (±0.2)	0.709
AQI	51.6 (±36.2)	0.073
PM2.5 (μg/m^3^)	31.8 (±28.9)	0.356
PM10 (μg/m^3^)	62.4 (±42.8)	0.139
CO (μg/m^3^)	0.6 (±0.3)	0.911
NO2 (μg/m^3^)	33.0 (±17.0)	0.137
SO2 (μg/m^3^)	8.3 (±4.1)	0.213
O3 (μg/m^3^)	92.8 (±36.3)	0.853
Treatment method for the aortic root		<0.001
Ascending aortic replacement	289 (57.2%)	
Selective Sinus Replacement	4 (0.8%)	
Wheats	5 (1.0%)	
David	4 (0.8%)	
Bentall	174 (34.5%)	
Ascending aortic replacement with coronary artery bypass grafting	10 (2.0%)	
Bentall with coronary artery bypass grafting	19 (3.8%)	
Treatment method for the aortic arch		<0.001
No arch intervention	39 (7.7%)	
Total arch replacement	459 (90.9%)	
Partial arch replacement	6 (1.2%)	
Debranching	1 (0.2%)	
Intraoperative blood transfusion (ml)	3942.4 (±1816.9)	<0.001
Duration of extracorporeal circulation (min)	222.7 (±94.1)	<0.001
Duration of aortic occlusion (min)	124.9 (±40.9)	<0.001
Duration of deep low-temperature shutdown cycle (min)	19.7 (±6.5)	<0.001
Duration of ventilator use (h)	180.5 (±330.6)	0.00053
Surgical duration (min)	452.7 (±134.9)	/
Male	457.9 (±144.3)	
Female	438.0 (±124.3)	

The statistical analysis of baseline characteristics and their associations with operative duration is summarized in Table 1. Significant demographic features included Age (*p* = 0.0096) and Weight (*p* = 0.023), while others were non-significant. Among comorbidities, Lung disease (*p* = 0.0129), Loss of consciousness (*p* = 0.00038), and Aortic valve insufficiency (*p* < 0.001) were important features. Laboratory findings highlighted White blood cells, Hemoglobin, C-reactive protein, Albumin, Troponin I or T, and D-dimer as significant correlates. Surgical related features, including Surgery Preparation Time, Intraoperative blood transfusion, Duration of extracorporeal circulation, Duration of aortic occlusion, and Duration of deep low-temperature shutdown cycle, were highly significant (all *p* < 0.001), while climate features showed no associations. Overall, operative duration was primarily influenced by patient condition and intraoperative management.

### 3.2 Surgical strategies and operative outcomes

A total of 505 patients were operated on by three different surgeons. As shown in Tab. S3, the distribution of cases was 118 (23.4%) for Surgeon A, 161 (31.9%) for Surgeon B, and 226 (44.7%) for Surgeon C. The mean surgical durations were 449.12 ± 144.44 min, 449.74 ± 104.85 min, and 456.64 ± 138.95 min, respectively. Statistical analysis showed no significant difference in surgical duration among the three surgeons (*p* = 0.828), indicating that surgical duration was not substantially influenced by the operating surgeon.

Surgical duration differed significantly across treatment methods for the aortic root (*p* < 0.01). In Tab. S4, aortic valve replacement (*n =* 198, 39.3%) had a mean duration of 474.1 ± 141.0 min, with Wheats (461.2 ± 25.5 min), Bentall (468.5 ± 133.0 min), and Bentall with coronary artery bypass grafting (528.8 ± 178.1 min). Valve-sparing aortic root replacement (*n =* 307, 60.7%) were associated with shorter operative times (438.9 ± 114.8 min), including ascending aortic replacement (436.9 ± 109.4 min), selective sinus replacement (464.3 ± 16.9 min), ascending aortic replacement with coronary artery bypass grafting (447.5 ± 38.5 min) and David (537.2 ± 49.2 min).

Surgical duration varied significantly across different treatment methods for the aortic arch (*p* < 0.01). Tab. S5 shows that total arch replacement was the predominant strategy (*n =* 459, 90.9%), with a mean duration of 459.2 ± 126.7 min. Among these, FET procedures (*n =* 411, 81.4%) averaged 465.7 ± 127.5 min, whereas non-FET procedures (*n =* 48, 9.5%) averaged 403.5 ± 118.0 min. No arch intervention cases (*n =* 39, 7.7%) had a mean duration of 384.2 ± 93.9 min. Partial arch replacement (*n =* 6, 1.2%) had a mean duration of 406.2 ± 40.9 min, and debranching (*n =* 1, 0.2%) required 419.6 min.

A significant positive correlation was identified between aortic occlusion time and operative duration (r = 0.64, *p* < 0.01). As shown in Supplementary Figure S1, patients with longer aortic occlusion times generally experienced prolonged surgical durations. Linear regression analysis further indicated a clear upward trend, suggesting that surgical duration increased proportionally with the extension of aortic occlusion time.

### 3.3 Predictive performance of ML models

To achieve accurate prediction of surgical duration for TAAD patients, this study constructed and compared the performance of 11 mainstream machine learning regression models, including Linear Regression, Elastic Net, Decision Tree Regression, Random Forest Regression, ExtraTrees Regression, Gradient Boosting Regression, XGBoost, CatBoost, AdaBoost, Support Vector Regression, and K-Nearest Neighbors Regression. The model evaluation indicators included the coefficient of determination (R^2^), root mean square error (RMSE), and mean absolute error (MAE).

**Figure 3:**
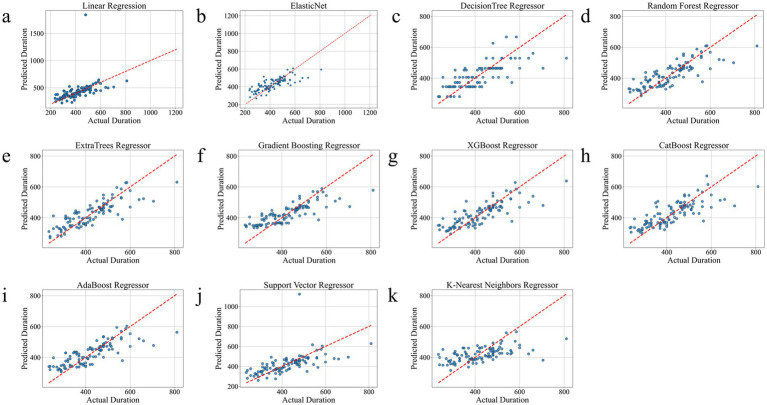
Comparison chart of the real and predicted effects of all models on the test set. **(a)**, Linear Regression. **(b)**, Elastic Net. **(c)**, Decision Tree Regressor. **(d)**, Random Forest Regressor. **(e)**, ExtraTrees Regressor. **(f)**, Gradient Boosting Regressor. **(g)**, XGBoost Regressor. **(h)**, CatBoost Regressor. **(i)**, AdaBoost Regressor. **(j)**, Support Vector Regressor. **(k)**, K-Nearest Neighbors Regressor.

**Figure 4:**
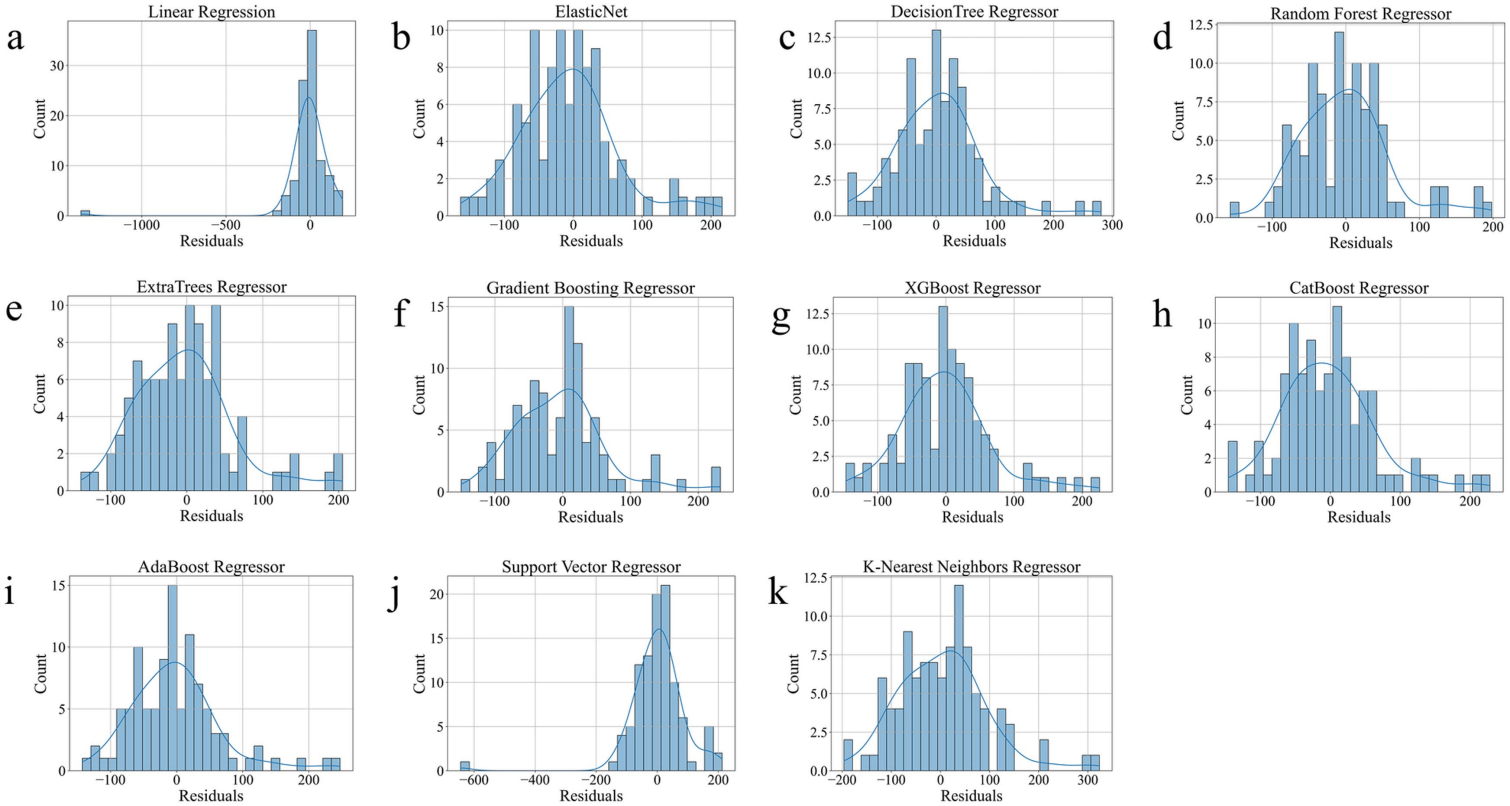
Residual Distribution of all models on the test set. **(a)**, Linear Regression. **(b)**, Elastic Net. **(c)**, Decision Tree Regressor. **(d)**, Random Forest Regressor. **(e)**, ExtraTrees Regressor. **(f)**, Gradient Boosting Regressor. **(g)**, XGBoost Regressor. **(h)**, CatBoost Regressor. **(i)**, AdaBoost Regressor. **(j)**, Support Vector Regressor. **(k)**, K-Nearest Neighbors Regressor.

**Figure 5:**
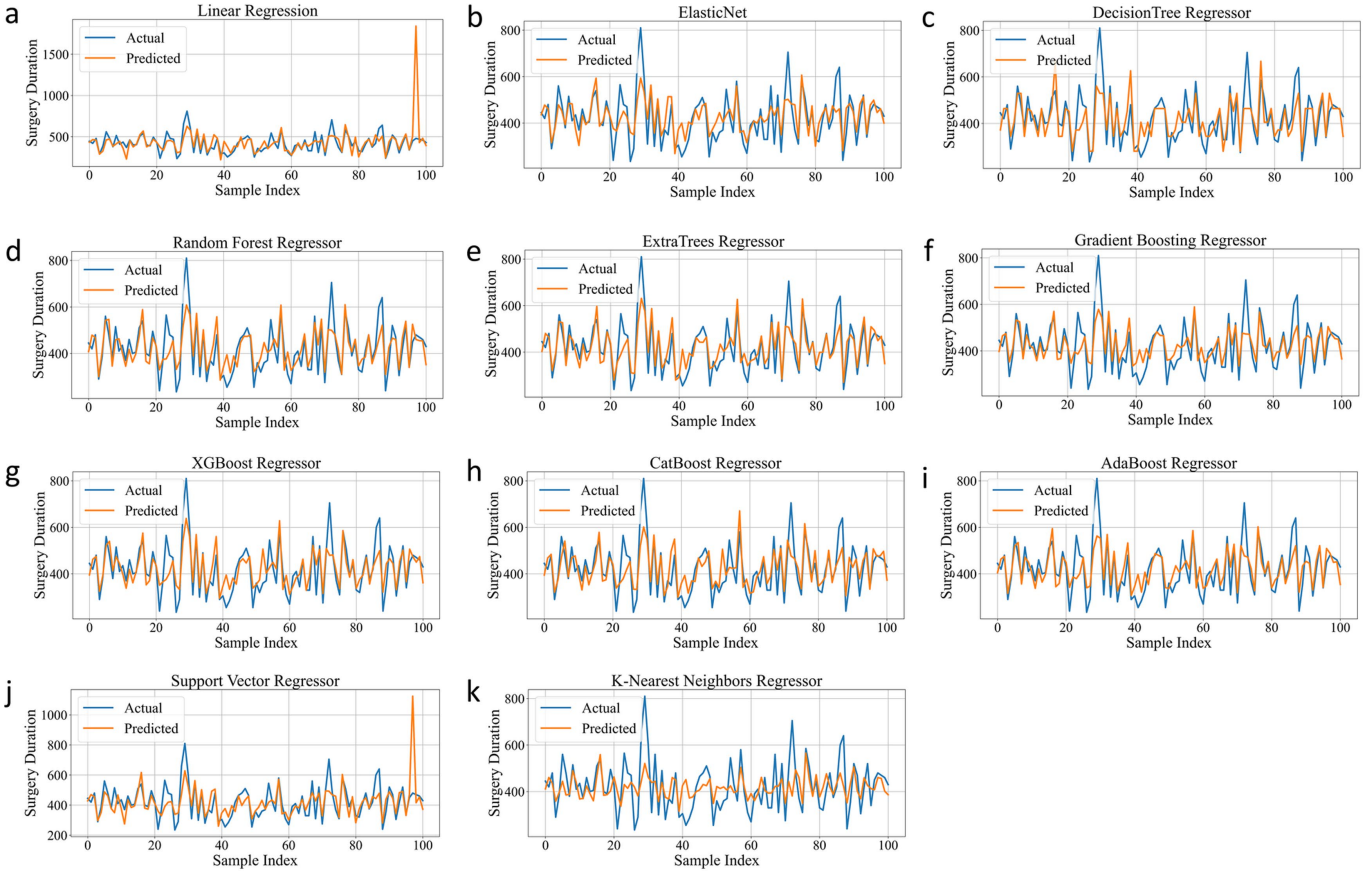
Prediction Trend of all models on the test set. **(a)**, Linear Regression. **(b)**, Elastic Net. **(c)**, Decision Tree Regressor. **(d)**, Random Forest Regressor. **(e)**, ExtraTrees Regressor. **(f)**, Gradient Boosting Regressor. **(g)**, XGBoost Regressor. **(h)**, CatBoost Regressor. **(i)**, AdaBoost Regressor. **(j)**, Support Vector Regressor. **(k)**, K-Nearest Neighbors Regressor.

As shown in Table 2, ExtraTrees Regressor (configured with n_estimators = 100, max_depth = 10, min_samples_split = 2, and min_samples_leaf = 2) achieved the best performance among all models on the test set, with an R^2^ of 0.7101, indicating that it explained approximately 71.01% of the variability in surgical duration. Additionally, its MAE was 43.54 min and RMSE was 59.42 min, both of which are the lowest among all models. The scatter plot comparing the actual and predicted values of the regression model as shown in Figure 3, further supports these findings. The predicted points of the ExtraTrees regressor were densely distributed around the ideal fitting line (y = x), with the smallest degree of deviation, indicating that the model achieved high fitting and consistency for surgical duration. The residual distribution shown in Figure 4 demonstrated that the residuals of the ExtraTrees model were approximately normally distributed, with most values concentrated around zero and without obvious skewness or heteroscedasticity, suggesting stable predictive performance across different time periods and strong generalization ability. As shown in Figure 5, the comparison chart reflected that the predicted curve generated by ExtraTrees closely followed the fluctuation trend of the actual surgical duration, accurately capturing multiple peaks and troughs, which demonstrates robust responsiveness to dynamic changes. In contrast, although XGBoost Regressor (R^2^ = 0.6219, MAE = 49.33, RMSE = 68.36) and CatBoost Regressor (R^2^ = 0.6285) also showed good performance, their performance in residual distribution and trend fitting were relatively limited compared to ExtraTrees, indicating reduced stability in handling clinical data with high noise and complex features. The traditional linear regression (R^2^ = −1.1302) and SVR (R^2^ = −0.3017) exhibited the poorest performance, as they failed to capture nonlinear patterns and were unsuitable for this task. As shown in Tab. S6, the ExtraTrees Regressor again achieved the best performance on the validation set (R^2^ = 0.7574, MAE = 46.19 min, RMSE = 58.60 min), surpassing all other models. Supplementary Figures S2–S4 confirmed its superior fitting, stable residual distribution, and close alignment of predicted with actual values, supporting strong robustness and generalization. Overall, ExtraTrees Regressor performed the best in this research task and can be used as the optimal model for predicting the surgical duration of TAAD patients.

**Table 2:** Model performance on the test set.

Model	R^2^	MAE	RMSE
Linear Regression	−1.1302	67.31	159.31
Elastic Net	0.5421	55.19	72.55
Decision Tree Regressor	0.5501	55.63	72.15
Random Forest Regressor	0.6415	49.36	65.74
ExtraTrees Regressor	0.7101	43.54	59.42
Gradient Boosting Regressor	0.5954	54.29	70.28
XGBoost Regressor	0.6219	49.33	68.36
CatBoost Regressor	0.6285	51.65	66.29
AdaBoost Regressor	0.6003	51.71	70.79
Support Vector Regressor	−0.3017	66.32	129.77
K-Nearest Neighbors Regressor	0.2914	74.01	94.65

### 3.4 Feature importance

To further explore the decision-making mechanism of the ExtraTrees Regressor model in predicting the surgical duration of patients with TAAD, this study applied the SHapley Additive exPlanations (SHAP) method to analyze the feature contributions and visualized them using beeswarm and bar plots.

**Figure 6:**
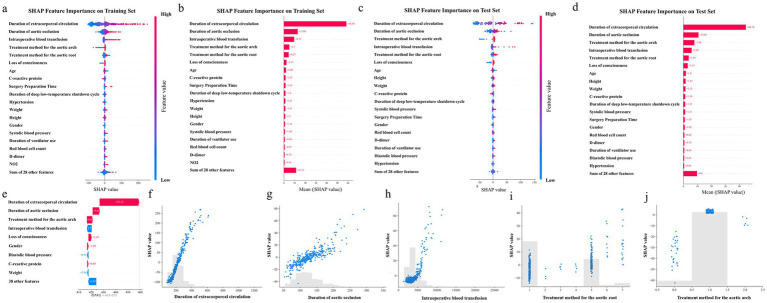
Top 20 feature importance. **(a)**, SHAP Beeswarm plot of the training set. **(b)**, SHAP Bar plot of the training set. **(c)**, SHAP Beeswarm plot of the test set. **(d)**, SHAP Bar plot of the test set. **(e)**, SHAP plot for a case study from the test set. **(f)**, SHAP value for Duration of extracorporeal circulation. **(g)**, SHAP value for Duration of aortic occlusion. **(h)**, SHAP value for Intraoperative blood transfusion. **(i)**, SHAP value for Treatment method for the aortic root. **(j)**, SHAP value for Treatment method for the aortic arch. Each point represents a patient sample, and the colors from blue to red indicate the feature values from low to high.

As shown in Figure 6a, the duration of extracorporeal circulation had the most significant positive impact on the model output, with high values (red) generally increasing the predicted surgical duration. Intraoperative blood transfusion and duration of aortic occlusion also significantly prolonged the surgical time, indicating that the intensity of intraoperative intervention is closely related to the duration of surgery. Other features, such as treatment method for the aortic root and arch, loss of consciousness, and C-reactive protein, also showed a certain degree of positive contribution, indicating the identifiable importance of the patient’s pathological status and surgical complexity in the model. Figure 6b quantifies the average impact of each variable on the overall predictions. The average SHAP value of extracorporeal circulation time was 58.58, making it the most influential feature. Duration of aortic occlusion (13.08) and intraoperative blood transfusion (9.72) followed, emphasizing the importance of intraoperative physiological load in predicting surgical duration. The treatment strategies for the aortic arch and root were also among the top contributors, highlighting that the choice of surgical approach and operational complexity were key factors affecting the duration of surgery. In addition, patient baseline factors such as age, weight, and history of hypertension also have moderate impact, while variables like gender, and height contribute relatively less. Figures 5d, 6c show the corresponding analysis in the test set, which were largely consistent with the training set. Additionally, the case study presented in Figure 6e confirmed that extracorporeal circulation time remained the dominant factor, while the relative contributions of other features aligned with the overall trend.

Figures 6f–j provide detailed SHAP scatter plots for the top five most influential features. As shown in Figure 6f, the duration of extracorporeal circulation exhibited a positive correlation with SHAP values, indicating that longer extracorporeal circulation generally contributed to increased predicted surgical durations. A similar trend was observed in Figure 6g, where the duration of aortic occlusion showed a linear increase in SHAP value, supporting its direct time-consuming nature during surgical manipulation. In Figure 6h, when intraoperative blood transfusion volume was relatively low (<3,000 mL), its impact on surgical duration was minimal. In the high transfusion range, SHAP values increased rapidly, suggesting that massive bleeding was associated with more complex intraoperative procedures. Figures 6i,j depict the influence of aortic root and aortic arch treatment strategies, respectively. Both the Bentall procedure and the Full Bow approach showed substantially positive SHAP contributions, indicating that these complex surgical techniques were consistently linked to extended operative times in the model’s interpretation.

Overall, the SHAP analysis results were highly consistent with clinical cognition, which enhanced the interpretability of the model and supports its practical application value in preoperative risk assessment and surgical planning.

## 4 Discussion

### 4.1 Comparison with the traditional methods

In traditional surgical duration prediction, common methods have included linear regression models and estimation based on a doctor’s experience. Linear models are constrained by the assumption of linear relationships between variables, making it difficult to capture complex nonlinear interactions and individual differences during surgery (40, 41). Prediction based on clinical experience is highly subjective and influenced by factors such as surgeon experience and patient anatomy, often resulting in significant errors (42, 43), especially in complex diseases like TAAD, which involves substantial variations in surgical pathways. In contrast, machine learning algorithms have demonstrated superior predictive abilities in multiple clinical studies due to their nonlinear modeling capabilities and advantages in processing high-dimensional, multimodal inputs (44). For example, Martinez found that ensemble learning models significantly improved the accuracy of surgical duration prediction (45).

### 4.2 Impact of surgical strategies on surgical duration

The similarity in operative times across different surgeons suggests a high level of consistency in surgical practice within this center. This finding reflects the impact of standardized surgical training, which helps to minimize inter-operator variability (46, 47).

The long operative duration observed for aortic valve repair in this cohort reflects its greater complexity, as repair procedures often require meticulous leaflet assessment, cusp resuspension or patch augmentation, and precise reconstruction of valve geometry to ensure competence (48). Valve replacement procedures place lower demands on leaflet preservation and therefore tend to be associated with shorter surgical duration. Both Wheat and Bentall belong to this category. However, Bentall procedures generally require longer root management because they involve concomitant coronary button reimplantation, in contrast to Wheat procedures (49).

In this study, the no arch intervention strategy was associated with the shortest surgical duration, as no additional arch reconstruction was required. In total arch replacement, the frozen elephant trunk (FET) technique requires additional steps for stent deployment and distal anastomosis in the descending aorta (50). Although the circulatory arrest time for these maneuvers is only prolonged by a few minutes compared with non-FET procedures (51), the associated increase in bleeding risk often results in a substantially longer hemostasis phase (52). The results of this study demonstrated that the average operative duration in FET cases was extended by nearly 1 h compared with non-FET arch replacement. Partial arch replacement showed shorter surgical duration than total arch replacement. This approach still required circulatory arrest under deep hypothermia, but operative time was reduced because supra-arch branches did not need reconstruction (53). Debranching procedures required no deep hypothermic circulatory arrest, and their operative times were between those of full arch and right half arch replacement (54).

Prolonged aortic occlusion time was closely associated with increased operative duration. This relationship reflects not only the extended time required for complex root and arch reconstruction but also the additional period needed for myocardial protection and meticulous hemostasis (55). Longer occlusion times have been linked to impaired coagulation and greater intraoperative blood loss, which further prolong surgical procedures (56).

### 4.3 Analysis of model performance

Among the multiple regression models applied in this study to predict the surgical duration of TAAD patients, traditional linear regression, support vector regression (SVR), and K-nearest neighbor (KNN) generally performed poorly. The main reason is that such models are unable to fully capture the multivariate interactions inherent in highly nonlinear and high-dimensional clinical data. The linear model assumes linear relationship between variables and is thus ineffective in addressing multifactorial problems (57, 58). Although SVR is theoretically suitable for nonlinear modeling, its performance is highly dependent on kernel selection and parameter tuning, making it prone to underfitting or overfitting (59, 60). The KNN algorithm is more sensitive to outliers and feature scaling, and tends to suffer from the “curse of dimensionality” in high-dimensional feature spaces, which substantially compromises predictive accuracy (61). In contrast, ensemble models are better able to capture nonlinear interactions between variables without relying on feature scaling or distributional assumptions, making them more robust to variable scales and types (57, 62). The ExtraTrees Regressor employs an extremely randomized partitioning strategy, selecting both features and thresholds at random during tree construction (63). This highly decorrelated strategy effectively reduces model variance and enhances generalization ability (64). Furthermore, ExtraTrees trains each tree using full sample set, which reduces model bias to some extent and helps better exploit available sample information (65).

In this study, ExtraTrees demonstrated superior performance in predicting the duration of TAAD surgery, confirming its capacity for modeling multifactorial, nonlinear, and interaction-rich problems. Overall, compared with traditional regression models, ensemble learning methods are more suitable for the complex prediction task of TAAD surgery duration.

### 4.4 Characteristics and clinical relevance

This study identified the top five key factors affecting the surgical duration of TAAD patients using SHAP value analysis (66): Duration of extracorporeal circulation, Duration of aortic occlusion, Intraoperative blood transfusion, Treatment method for the aortic arch, and Treatment method for the aortic root. These factors directly reflected the surgical complexity and were closely related clinically to operative duration.

Duration of extracorporeal circulation and duration of aortic occlusion were direct measures of intraoperative process duration. They correlated with prolonged total operative time, especially in surgeries involving more extensive reconstruction or older patients who require tailored perfusion strategies (67, 68). Intraoperative blood transfusion was frequently observed in cases with significant bleeding or coagulation disturbances and was consistently associated with longer operative times. This reflected the additional hemostatic interventions and procedural complexity required in these settings, as reported in recent TAAD cohorts (69). In TAAD surgery, the arch strategy and root strategy were closely linked to operative duration, serving as indicators of procedural complexity. Total arch replacement was repeatedly shown to involve substantially longer cardiopulmonary bypass and circulatory arrest times compared with hemiarch replacement (70). Likewise, the choice of aortic root procedure affected ischemic and perfusion times, with valve-sparing root replacement generally associated with a longer operative duration than composite root replacement (71, 72). These relationships should be interpreted as indicators of procedural complexity rather than independent preoperative causal determinants. They highlighted how surgical techniques and intraoperative conditions influence operative time, providing mechanistic insight into why certain TAAD procedures were substantially longer and technically more demanding.

In addition to the five main factors mentioned above, this study also included variables such as patient age, gender, physical indicators, symptom presentation (including preoperative loss of consciousness), laboratory indicators (such as C-reactive protein and D-dimer), and surgery preparation time. Compared with the dominant intraoperative variables, these factors demonstrated substantially lower SHAP values, indicating a relatively limited contribution to predicting of surgical duration. This indicates that the duration of surgery was primarily influenced by the technical difficulty and process events of the surgery itself, while the patient’s basic condition and preoperative preparation may affect the overall surgical risk but have a relatively small direct impact on the duration of surgery (73).

Although Marfan syndrome was not among the features with the highest SHAP values, the broader category of heritable thoracic aortic diseases, which it exemplifies, warrants dedicated discussion due to its clinical and genetic significance.

Heritable Thoracic Aortic Disease (HTAD) encompasses a heterogeneous group of conditions, including syndromic forms such as Marfan syndrome and Loeys–Dietz syndrome, as well as congenital abnormalities like bicuspid aortic valve (BAV) and coarctation of the aorta. Accumulating evidence suggests that the presence of HTAD increases surgical complexity and consequently prolongs operative duration in TAAD (74). For instance, BAV patients require simultaneous replacement of the aortic valve during valve disease surgery, resulting in longer surgical time (75). Coarctation of the aorta often results in deformities of the aortic arch and descending aorta, which increase the technical difficulty of intraoperative management and consequently prolong surgical time (76). Marfan and Loeys–Dietz are characterized by fragile aortic tissues, leading to more complex root and arch reconstructions that likely extend surgical duration (77, 78). It is important to consider HTAD subtypes, as their distinct features significantly influence intraoperative complexity and contribute to prolonged operative duration in TAAD.

### 4.5 Limitations

The data in this study mainly comes from a single center, and the sample size is relatively limited, which may lead to a decrease in the predictive performance of the model on independent external samples or regional bias. It is recommended that future research adopt larger-scale, multi-center, prospective designs. Another limitation is that several of the top features identified are only available intraoperatively, which restricts the model’s applicability for preoperative prediction. Nevertheless, the results underscore the critical influence of intraoperative complexity on operative duration and indicate potential targets for procedural optimization and resource allocation. Accordingly, our model should be interpreted as providing explanatory insights into surgical complexity rather than functioning as a direct preoperative prediction tool, while future studies are needed to incorporate richer preoperative data to improve predictive utility. Moreover, this study did not include an external validation cohort, which limits the generalizability of the findings. Future work should incorporate multi-center datasets for prospective validation.

## 5 Conclusion

The study constructed and validated 11 regression models for analyzing the duration of TAAD surgery, using data from Qingdao University Affiliated Hospital. The ExtraTrees Regressor model has the best performance (R^2^ = 0.7101, MAE = 43.54, RMSE = 59.42). Compared with traditional prediction methods, machine learning models can integrate multidimensional features such as demographics, clinical indicators, environment, and surgical types, and significantly improve prediction performance by learning complex nonlinear relationships and interactions. Surgical duration in TAAD is varied substantially with procedural strategies. Complex root and arch management strategies are associated with prolonged operative duration, and extended aortic occlusion time further contributes to this increase. The model identified Duration of extracorporeal circulation, Intraoperative blood transfusion, Duration of aortic occlusion, Treatment method for the aortic arch, and Treatment method for the aortic root as the five most influential factors affecting operative duration, all of which warrant careful consideration by cardiac surgeons during preoperative planning. The impact of Marfan syndrome on surgical duration is not significant in this study. Nonetheless, HTAD is characterized by underlying connective tissue abnormalities that can increase intraoperative technical challenges, and its potential influence on surgical outcomes warrants further investigation in larger cohorts. This study provides an explanatory analysis of factors influencing operative duration in Stanford type A aortic dissection. The inclusion of intraoperative variables offers important insights into procedural complexity and clinical management, although it limits immediate preoperative application. Future research will focus on models based solely on preoperative features with external validation to enable real-time prediction and broader clinical use.

## Data Availability

The dataset used in this study is available from the corresponding author upon reasonable request and with approval from the Ethics Committee of Qingdao University.
