# Supplementary material for: Machine learning-based analysis of factors influencing surgical duration in type A aortic dissection
Source: Front Public Health. 2025 Oct 31;13:1682339. doi: 10.3389/fpubh.2025.1682339 (PMC12615382; doi:10.3389/fpubh.2025.1682339)
Supplement: Supplementary file 1 [file Supplementary_file_1.docx]

**Supplementary materials**

**1. Feature encoding**

**1.1 Gender**

Male 1, female 2.

**1.2 Comorbidities**

Hypertension/Diabetes/Heart disease/Loss of consciousness/Lung disease: yes 0, no 1.

Aortic valve insufficiency: Mild 1, Mild to Moderate 2, Moderate 3, Moderate to Severe 4, Severe 5.

Marfan syndrome: no 0, yes 1.

**1.3 Treatment method for the aortic root**

Ascending aortic replacement 1, Selective Sinus Replacement 2, Wheats 3, David 4, Bentall 5, Ascending aortic replacement with coronary artery bypass grafting 6, Bentall with coronary artery bypass grafting 7.

**1.4 Treatment method for the aortic arch**

No arch intervention 0, Total arch replacement 1, Partial arch replacement 2, Debranching 3.

**2. Sample size calculation**

To justify the adequacy of the available sample, a precision-based framework was applied. For a continuous outcome such as operative duration, the minimum sample size required to achieve a desired two-sided 95% confidence interval half-width $d$ is expressed as:

$$n\geq{( \frac{Z_{1-\alpha/2}\sigma}{d})}^{2}$$

where $\sigma$ is the population standard deviation and $Z_{1-\alpha/2}$ is the standard normal quantile.

**Tab. S1 Variables and their corresponding units.**

| **Variable** | **Unit** |
| --- | --- |
| Gender | / |
| Age | years |
| Height | cm |
| Weight | kg |
| Heart rate | beats per minute (bpm) |
| Body temperature | °C |
| Systolic blood pressure | mmHg |
| Diastolic blood pressure | mmHg |
| Hypertension | / |
| Diabetes | / |
| Heart disease | / |
| Lung disease | / |
| Marfan syndrome | / |
| Loss of consciousness | / |
| Aortic valve insufficiency | / |
| White blood cells | ×10⁹/L |
| Red blood cells | ×10⁹/L |
| Hemoglobin | g/L |
| Platelets | ×10⁹/L |
| C-reactive protein | mg/L |
| Albumin | g/L |
| Alanine transaminase | U/L |
| Aspartate transaminase | U/L |
| Creatinine | umol/L |
| Troponin I or T | ng/mL |
| D-dimer | μg/L |
| Smoking history | / |
| Drinking history | / |
| Surgery Preparation Time | days |
| ICU stay days before surgery | days |
| Minimum temperature | °C |
| Maximum temperature | °C |
| Relative humidity | % |
| AQI | index |
| PM2.5 | μg/m³ |
| PM10 | μg/m³ |
| CO | μg/m³ |
| NO2 | μg/m³ |
| SO2 | μg/m³ |
| O3 | μg/m³ |
| Treatment method for the aortic root | / |
| Treatment method for the aortic arch | / |
| Intraoperative blood transfusion | mL |
| Duration of extracorporeal circulation | minutes |
| Duration of aortic occlusion | minutes |
| Duration of deep low-temperature shutdown cycle | minutes |
| Duration of ventilator use | hours |
| Surgical duration | minutes |

**Tab. S2 Baseline data of excluded patients.**

| **Characteristics** | **Mean/count (±SD/%)** |
| --- | --- |
| **Demographics** |  |
| Gender |  |
| *Male* | 9 (75%) |
| *Female* | 3 (25%) |
| Age | 56.3 (±10.8) |
| Height (cm) | 176.8 (±7.5) |
| Weight (kg) | 80.1 (±21.5) |
| Heart rate (bpm) | 79.1 (±20.3) |
| Body temperature (℃) | 36.5 (±0.2) |
| Systolic blood pressure (mmHg) | 138.7 (±28.12) |
| Diastolic blood pressure (mmHg) | 73.6 (±25.71) |
| **Comorbidities** |  |
| Hypertension | 7 (58.3%) |
| Diabetes | 10 (83.3%) |
| Heart disease | 10 (83.3%) |
| Lung disease | / |
| Marfan syndrome | / |
| Loss of consciousness | 2 (16.7 %) |
| Aortic valve insufficiency |  |
| *Mild* | 1 (8.3%) |
| *Mild to Moderate* | / |
| *Moderate* | / |
| *Moderate to Severe* | / |
| *Severe* | / |
| **Laboratory** |  |
| White blood cells (×10⁹/L) | / |
| Red blood cells (×10⁹/L) | / |
| Hemoglobin (g/L) | / |
| Platelets (×10⁹/L) | / |
| C-reactive protein (mg/L) | / |
| Albumin (g/L) | / |
| Alanine transaminase (U/L) | / |
| Aspartate transaminase (U/L) | / |
| Creatinine (umol/L) | / |
| Troponin I or T (ng/mL) | / |
| D-dimer (μg/L) | / |
| **Lifestyle Factors** |  |
| Smoking history | 4 (33.3 %) |
| Drinking history | 2 (16.7 %) |
| **Surgical related data** |  |
| Surgery Preparation Time (day) | / |
| ICU stay days before surgery (day) | / |
| Minimum temperature (℃) | / |
| Maximum temperature (℃) | / |
| Relative humidity | / |
| AQI | / |
| PM2.5 (μg/m³) | / |
| PM10 (μg/m³) | / |
| CO (μg/m³) | / |
| NO2 (μg/m³) | / |
| SO2 (μg/m³) | / |
| O3 (μg/m³) | / |
| Treatment method for the aortic root |  |
| *Ascending aortic replacement* | / |
| *Selective Sinus Replacement* | / |
| *Wheats* | / |
| *David* | / |
| *Bentall* | / |
| *Ascending aortic replacement with coronary artery bypass grafting* | / |
| *Bentall with coronary artery bypass grafting* | / |
| Treatment method for the aortic arch |  |
| *No arch intervention* | / |
| *Total arch replacement* | / |
| *Partial arch replacement* | / |
| *Debranching* | / |
| Intraoperative blood transfusion(ml) | / |
| Duration of extracorporeal circulation(min) | / |
| Duration of aortic occlusion(min) | / |
| Duration of deep low-temperature shutdown cycle(min) | / |
| Duration of ventilator use (h) | / |
| Surgical duration (min) | / |
| *Male* | / |
| *Female* | / |

**Tab. S3 Comparison of operative duration among three surgeons.**

| **Surgeon** | **n (%)** | **Surgical duration (Mean, ±SD)** | **P-value** |
| --- | --- | --- | --- |
| A | 118 (23.4%) | 449.12 (±144.44) |  |
| B | 161 (31.9%) | 449.74 (±104.85) | 0.828 |
| C | 226 (44.7%) | 456.64 (±138.95) |  |

**Tab. S4 Surgical duration across different treatment methods for the aortic root.**

| **Treatment method** | **n (%)** | **Surgical duration (Mean, ±SD)** | **P-value** |
| --- | --- | --- | --- |
| **Aortic valve replacement** | **198 (39.3%)** | **474.1 (±141.0)** | < 0.01 |
| Wheats | 5 (1.0%) | 461.2 (±25.5) |  |
| Bentall | 174 (34.5%) | 468.5 (±133.0) |  |
| Bentall with coronary artery bypass grafting | 19 (3.8%) | 528.8 (±178.1) |  |
| **Valve-sparing aortic root replacement** | **307(60.7%)** | **438.9 (±114.8)** |  |
| Ascending aortic replacement | 289 (57.2%) | 436.9 (±109.4) |  |
| Selective Sinus Replacement | 4 (0.8%) | 464.3 (±16.9) |  |
| Ascending aortic replacement with coronary artery bypass grafting | 10 (2.0%) | 447.5 (±38.5) |  |
| David | 4 (0.8%) | 537.2 (±49.2) |  |

**Tab. S5 Surgical duration across different treatment methods for the aortic arch.**

| **Treatment method** | **n (%)** | **Surgical duration (Mean, ±SD)** | **P-value** |
| --- | --- | --- | --- |
| **No arch intervention** | **39 (7.7%)** | **384.2 (±93.9)** | < 0.01 |
| **Total arch replacement** | **459 (90.9%)** | **459.2 (±126.7)** |  |
| FET | 411 (81.4%) | 465.7 (±127.5) |  |
| Non-FET | 48 (9.5%) | 403.5 (±118.0) |  |
| **Partial arch replacement** | **6 (1.2%)** | **406.2 (±40.9)** |  |
| **Debranching** | **1 (0.2%)** | **419.6 (±0)** |  |

**Tab. S6 Model performance on the validation set.**

| **Model** | **R²** | **MAE** | **RMSE** |
| --- | --- | --- | --- |
| Linear Regression | -1.0132 | 67.07 | 143.79 |
| Elastic Net | 0.5936 | 54.23 | 71.09 |
| Decision Tree Regressor | 0.5692 | 54.51 | 72.08 |
| Random Forest Regressor | 0.6723 | 49.11 | 63.92 |
| ExtraTrees Regressor | 0.7574 | 46.19 | 58.60 |
| Gradient Boosting Regressor | 0.6231 | 51.45 | 68.54 |
| XGBoost Regressor | 0.7037 | 48.19 | 66.10 |
| CatBoost Regressor | 0.6986 | 49.99 | 64.32 |
| AdaBoost Regressor | 0.6643 | 50.26 | 68.37 |
| Support Vector Regressor | -0.2235 | 60.82 | 107.97 |
| K-Nearest Neighbors Regressor | 0.3668 | 73.21 | 89.75 |


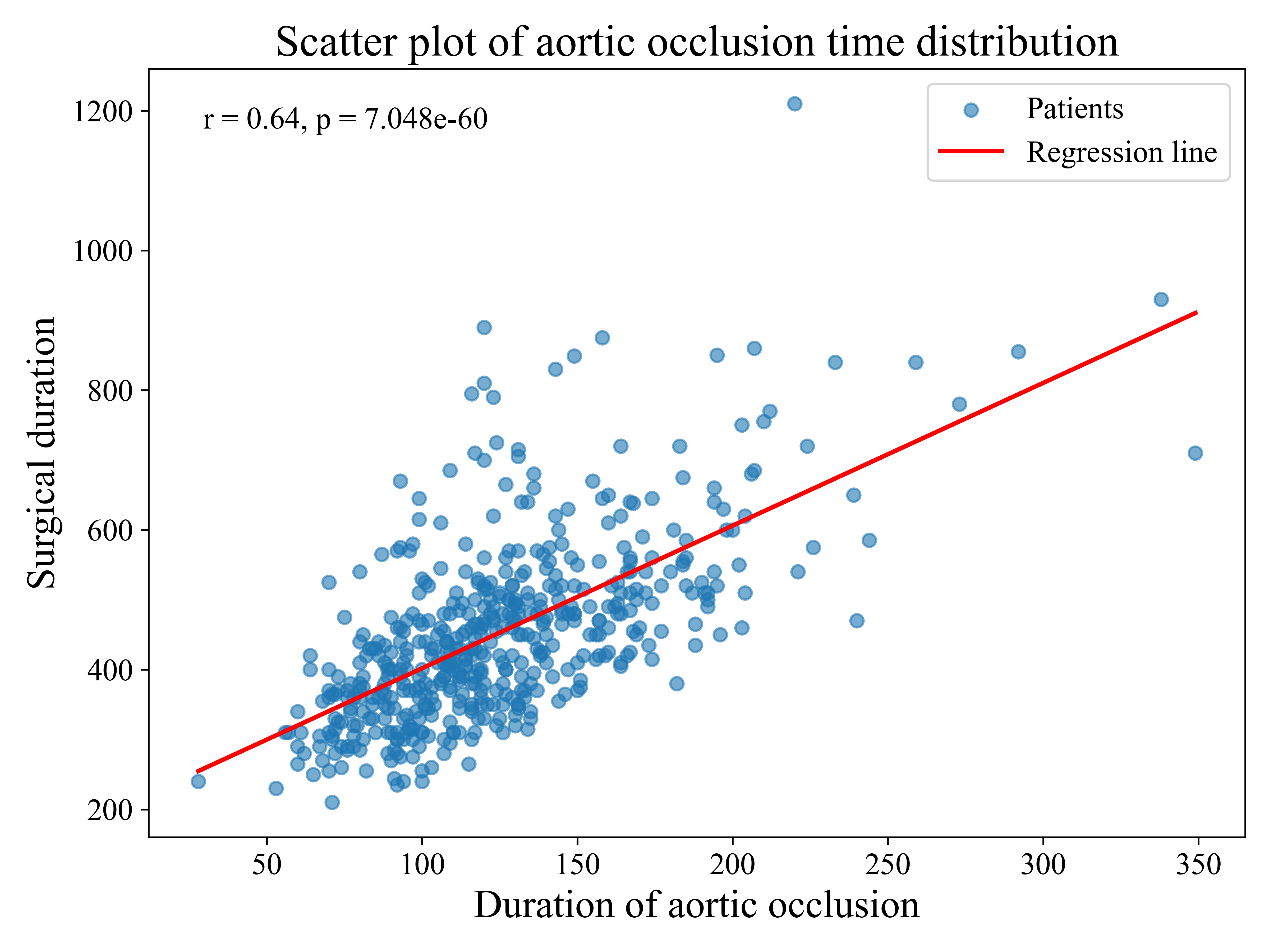


**Fig. S1 Scatter plot of aortic occlusion time distribution.**


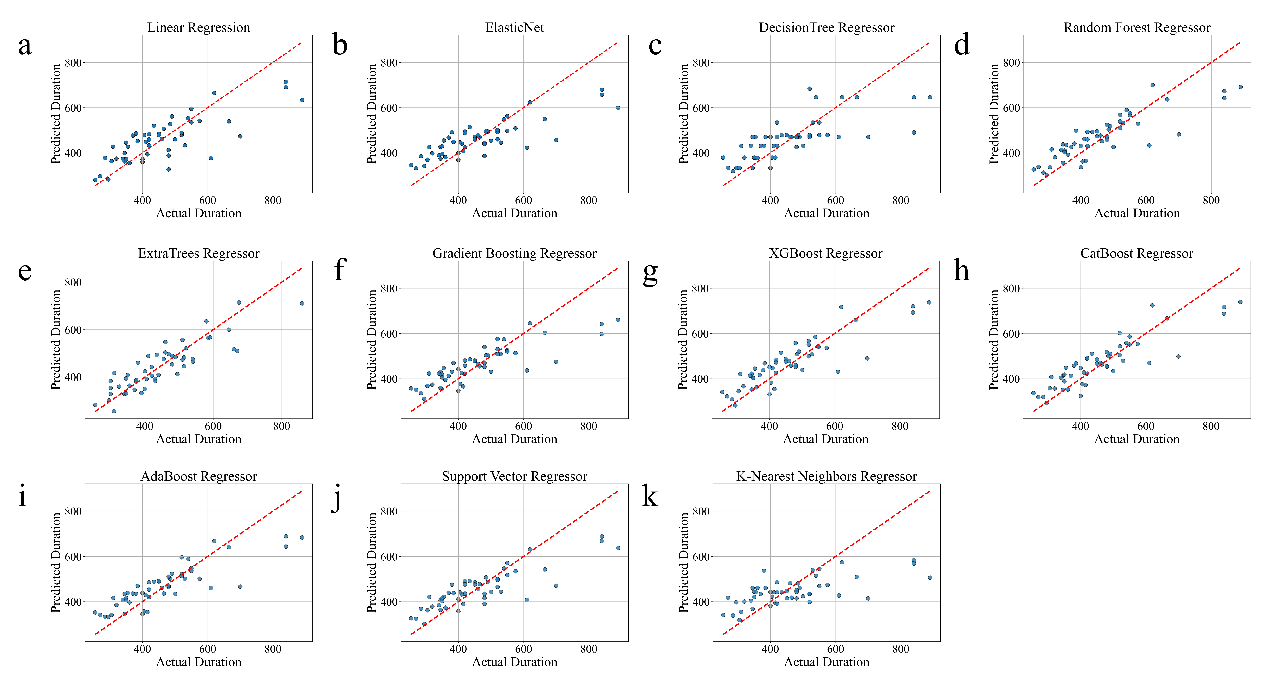


**Fig. S2 Comparison chart of actual and predictive performance of all models on the validation set.** a, Linear Regression. b, Elastic Net. c, Decision Tree Regressor. d, Random Forest Regressor. e, ExtraTrees Regressor. f, Gradient Boosting Regressor. g, XGBoost Regressor. h, CatBoost Regressor. i, AdaBoost Regressor. j, Support Vector Regressor. k, K-Nearest Neighbors Regressor.


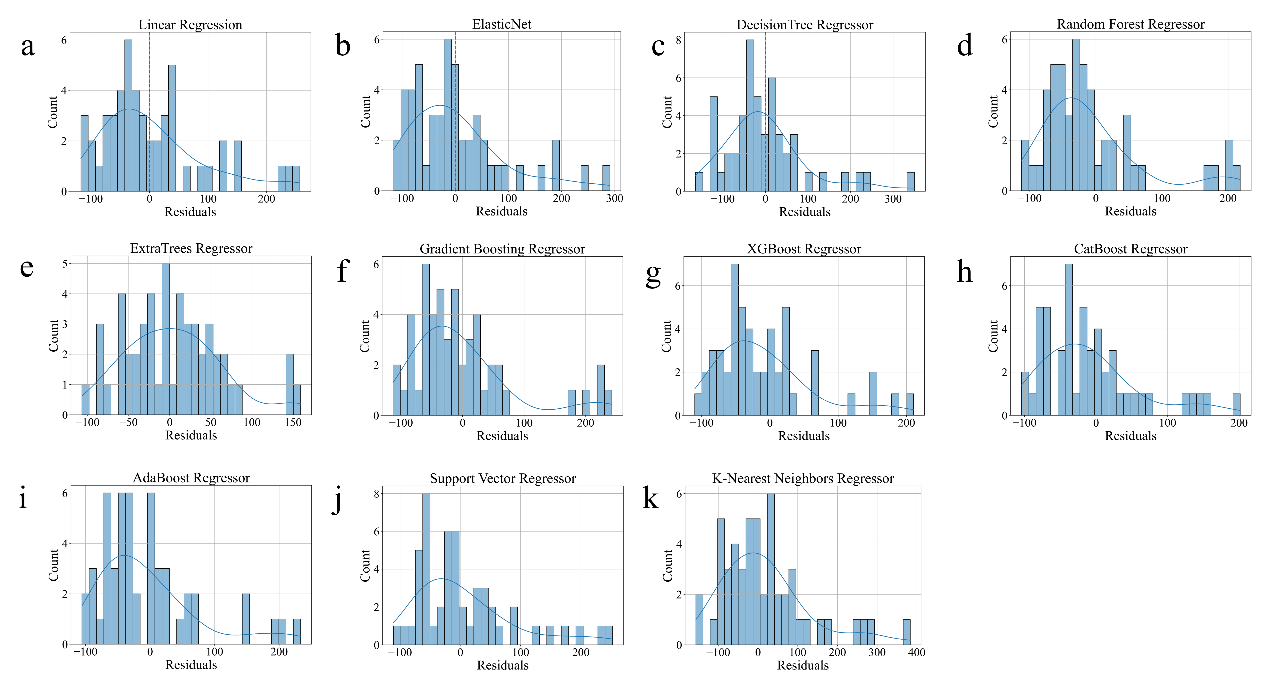


**Fig. S3 Residual Distribution of all models on the validation set.** a, Linear Regression. b, Elastic Net. c, Decision Tree Regressor. d, Random Forest Regressor. e, ExtraTrees Regressor. f, Gradient Boosting Regressor. g, XGBoost Regressor. h, CatBoost Regressor. i, AdaBoost Regressor. j, Support Vector Regressor. k, K-Nearest Neighbors Regressor.


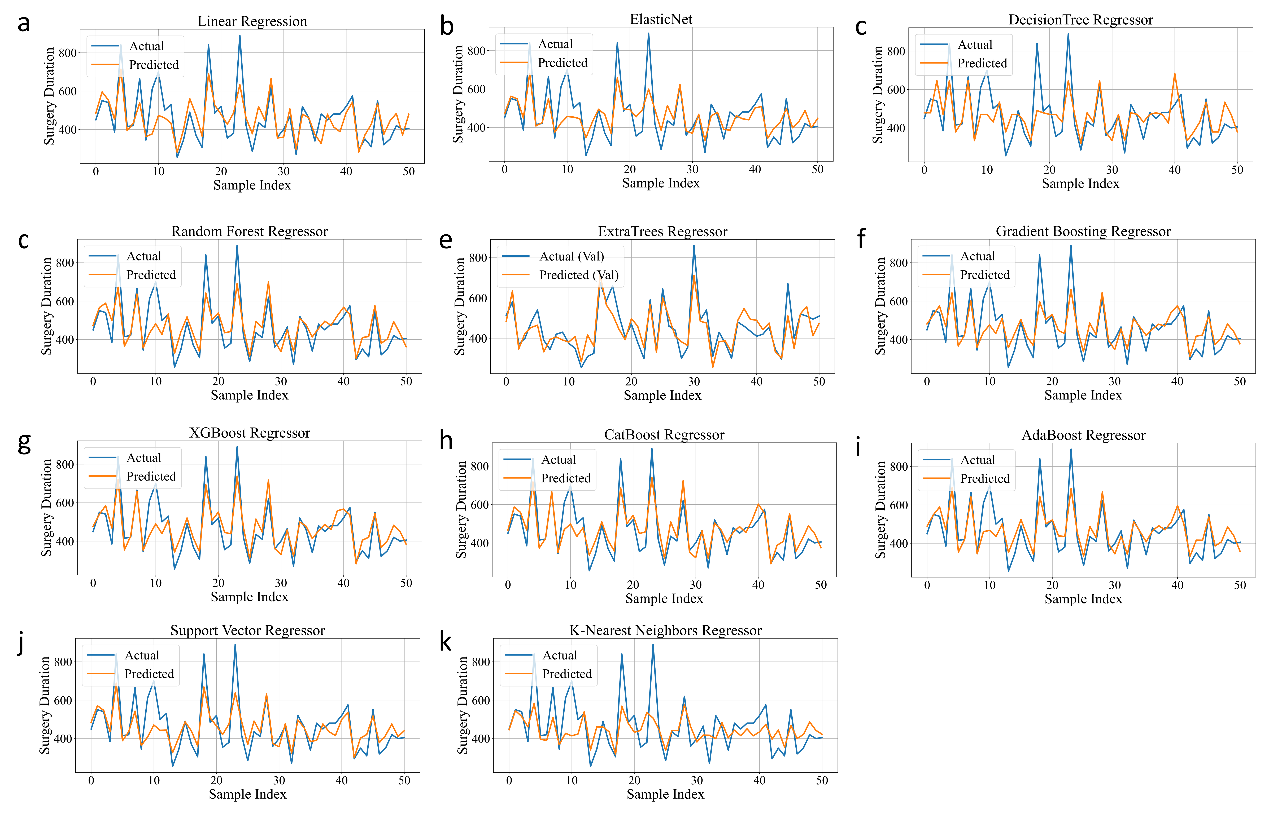


**Fig. S4 Prediction Trend of all models on the validation set.** a, Linear Regression. b, Elastic Net. c, Decision Tree Regressor. d, Random Forest Regressor. e, ExtraTrees Regressor. f, Gradient Boosting Regressor. g, XGBoost Regressor. h, CatBoost Regressor. i, AdaBoost Regressor. j, Support Vector Regressor. k, K-Nearest Neighbors Regressor.
